# Development and large volume production of extremely high current density YBa_2_Cu_3_O_7_ superconducting wires for fusion

**DOI:** 10.1038/s41598-021-81559-z

**Published:** 2021-01-22

**Authors:** A. Molodyk, S. Samoilenkov, A. Markelov, P. Degtyarenko, S. Lee, V. Petrykin, M. Gaifullin, A. Mankevich, A. Vavilov, B. Sorbom, J. Cheng, S. Garberg, L. Kesler, Z. Hartwig, S. Gavrilkin, A. Tsvetkov, T. Okada, S. Awaji, D. Abraimov, A. Francis, G. Bradford, D. Larbalestier, C. Senatore, M. Bonura, A. E. Pantoja, S. C. Wimbush, N. M. Strickland, A. Vasiliev

**Affiliations:** 1 S-Innovations, Moscow, Russia; 2 SuperOx, Moscow, Russia; 3 Joint Institute for High Temperature, Russian Academy of Sciences, Moscow, Russia; 4 SuperOx Japan, Kanagawa, Japan; 5 Commonwealth Fusion Systems, Cambridge, MA USA; 6 Massachusetts Institute of Technology, Cambridge, MA USA; 7 P.N. Lebedev Physics Institute, Russian Academy of Sciences, Moscow, Russia; 8 Institute for Materials Research, Tohoku University, Sendai, Japan; 9 National High Magnetic Field Laboratory, Florida State University, Tallahassee, FL USA; 10 University of Geneva, Geneva, Switzerland; 11 Robinson Research Institute, Victoria University of Wellington, Wellington, New Zealand; 12 National Research Centre “Kurchatov Institute”, Moscow, Russia; 13 Shubnikov Institute of Crystallography, Russian Academy of Sciences, Moscow, Russia; 14 Moscow Institute of Physics and Technology, Dolgoprudny, Russia

**Keywords:** Superconducting properties and materials, Superconducting properties and materials

## Abstract

The fusion power density produced in a tokamak is proportional to its magnetic field strength to the fourth power. Second-generation high temperature superconductor (2G HTS) wires demonstrate remarkable engineering current density (averaged over the full wire), *J*_*E*_, at very high magnetic fields, driving progress in fusion and other applications. The key challenge for HTS wires has been to offer an acceptable combination of high and consistent superconducting performance in high magnetic fields, high volume supply, and low price. Here we report a very high and reproducible *J*_*E*_ in practical HTS wires based on a simple YBa_2_Cu_3_O_7_ (YBCO) superconductor formulation with Y_2_O_3_ nanoparticles, which have been delivered in just nine months to a commercial fusion customer in the largest-volume order the HTS industry has seen to date. We demonstrate a novel YBCO superconductor formulation without the *c*-axis correlated nano-columnar defects that are widely believed to be prerequisite for high in-field performance. The simplicity of this new formulation allows robust and scalable manufacturing, providing, for the first time, large volumes of consistently high performance wire, and the economies of scale necessary to lower HTS wire prices to a level acceptable for fusion and ultimately for the widespread commercial adoption of HTS.

## Introduction

The discovery of high temperature superconductivity (HTS) in 1986^1^ generated great hope for widespread superconducting devices. Today we can see that it took more time and effort to develop HTS technology than many had anticipated. But that development brought about practical 2G HTS wires that now can revolutionise some of the most basic branches of the world’s economy. Among their many important uses, HTS wires enable very high magnetic fields with a relatively low power input. The 26.4 T all-HTS magnet^2^ and the 45.5 T hybrid HTS magnet^3^ are recent landmark achievements in this field. Perhaps the most significant impact HTS materials can make is in magnetic confinement fusion devices^4,5^. Fusion has the potential to rewrite the electric power landscape of mankind, solving rapidly accelerating climate issues and bringing affordable, non-polluting power to billions of people.

The progress of HTS technology has been especially impressive in the last decade, culminating recently in *J*_*E*_ values in excess of 1000 A/mm^2^ at 4.2 K in 18–20 T magnetic field^6,7^. While this is ample for some applications^8^, higher *J*_*E*_ opens new frontiers in magnets for fusion reactors^9,10^, particle accelerators^11–13^, magnetic resonance imaging^14^, nuclear magnetic resonance spectroscopy^15,16^ and space detectors^17^. For instance, a minimum engineering current density of 700 A/mm^2^ at 20 K, 20 T is essential for the magnet system of the prototype commercial fusion device SPARC^18^, which is being designed and constructed by a collaboration of MIT and Commonwealth Fusion Systems (CFS). This target was a significant challenge when first announced, because even the best laboratory samples could barely reach that performance. Moreover, all the above applications require large wire volumes, the SPARC device for example needing approximately 10,000 km of 4 mm wide wire. For most commercial applications of HTS, the wire cost is still too high for commercial viability and only robust, high-volume manufacturing can lower the price to a level amenable to the widespread commercial adoption of HTS^19^.

In this article we demonstrate very high engineering current density in practical 2G HTS wires based on a simple YBCO superconductor formulation with Y_2_O_3_ nanoparticles. We present a large and consistent dataset of critical current measurements independently performed in leading laboratories worldwide on wires delivered in large industrial volumes for a commercial fusion power customer.

## Concept

Our concept for developing HTS wire with high in-field performance suitable for large volume, low cost manufacturing was the following:

1. Select yttrium as the rare earth element in place of gadolinium or europium as commonly used in the 2G HTS wire industry. We choose yttrium because of its small ionic radius, which results in higher charge carrier (hole) density in the superconducting CuO_2_ planes and also because it confers a lower electronic anisotropy and higher irreversibility field^20^.
2. Employ uniformly distributed Y_2_O_3_ nanoparticles, native to YBCO, as vortex pinning centres, thus keeping the composition and microstructure simple to facilitate reproducible fabrication. This contrasts with the common approach of introducing extrinsic nano-columns aligned about the REBCO *c*-axis as pinning centres^21–25^, a proven challenge to industrial implementation^25–28^.
3. Take advantage of the very low neutron cross-section of Y of 1.28 b compared to those of Gd (49,000 b) and Eu (4570 b), which is particularly important for application in a fusion reactor.
4. Use thin substrate and deposit thick YBCO films, to further increase engineering current density.

## Experimental results

In 9 months, we manufactured over 300 km of 4 mm wide YBCO wire, delivering most of it to CFS. This has been the largest completed order in the history of the 2G HTS wire industry. We fabricated the wire on a strong Hastelloy C276 substrate with a buffer layer architecture based on MgO textured using ion beam assisted deposition and with the HTS layer grown by pulsed laser deposition (PLD), as explained in the Methods section. The YBCO layer contains uniformly distributed Y_2_O_3_ nanoparticles with (001) and (110) axial orientation, with the average density of 4000 ± 2000 μm^-2^. Microstructural analysis is presented in Supplementary Information.

### Superconducting properties in high magnetic field

**Figure 1:**
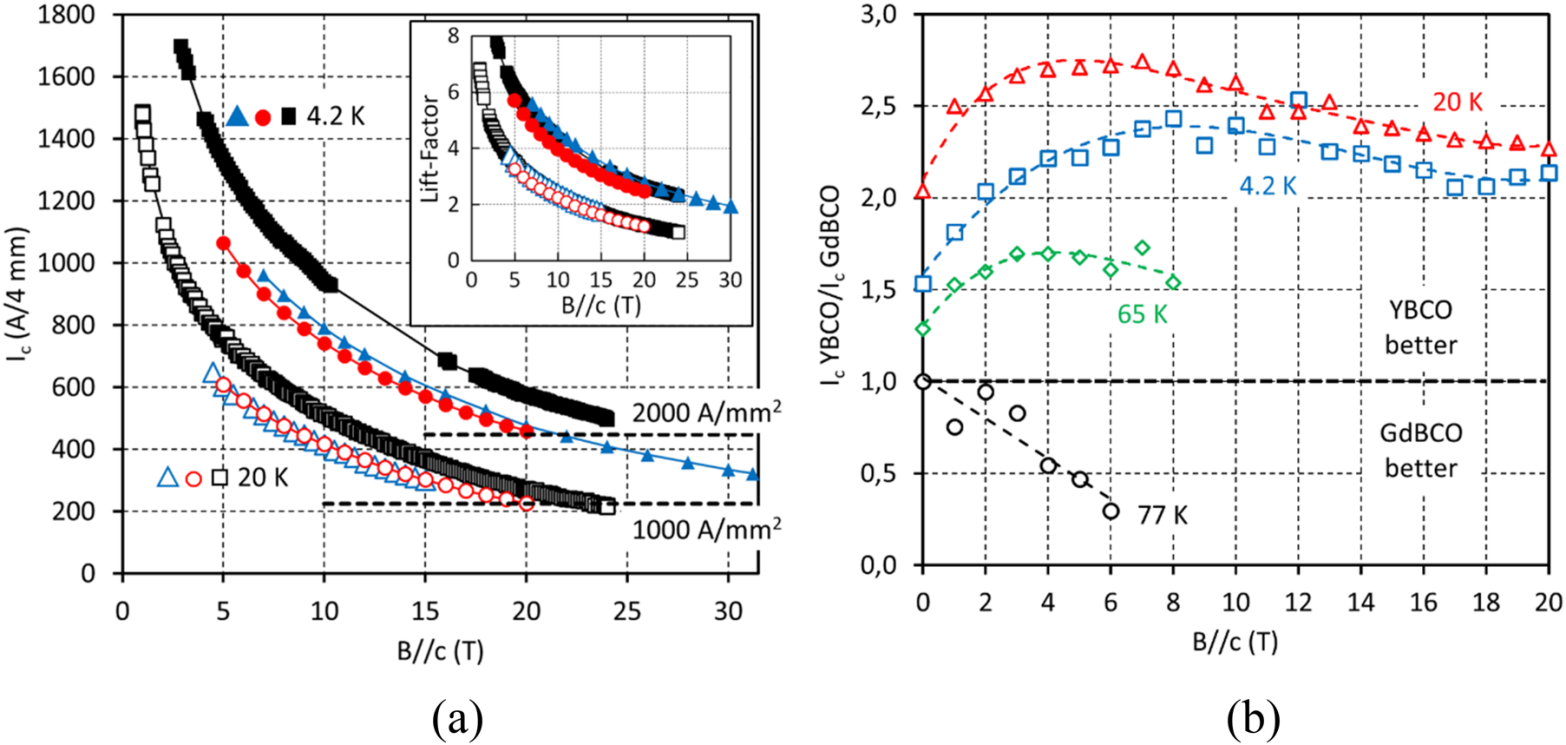
(**a**) Critical current, *I*_*c*_, in magnetic field (*B//c*) at 4.2 and 20 K of three YBCO wire samples measured at University of Geneva (red curves), Tohoku University (black curves) and the NHMFL at Florida State University (blue curves). The 1000 and 2000 A/mm^2^ marks for engineering current density, *J*_*E*_, are provided for wire on 40 μm substrate with 5 μm per side stabilising copper layer. In the inset: lift-factors based on 77 K, self-field *I*_*c*_ values. Very high values of *I*_*c*_ and *J*_*E*_ have been achieved. Although the *I*_*c*_ values in the three samples are noticeably different, the lift-factor values are very close, manifesting good process reproducibility and predictability of superconducting performance. (**b**) Ratios of *I*_*c*_ values of YBCO and GdBCO wires at 4.2, 20, 65, and 77 K in magnetic field. Wherever the ratio is greater than unity, the superconducting performance of YBCO is superior to that of GdBCO and vice versa. Dashed lines on the plot are provided for guidance only.

In Fig. 1a we present the superconducting performance at low temperature and high magnetic field oriented perpendicular to the wire surface (*B//c*) of three representative YBCO wires measured at high field facilities in Switzerland, Japan and the USA. The samples differed in substrate thickness: 40, 60 and 100 µm and YBCO layer thickness: 2.55, 2.82 and 3.28 µm, respectively. In all three samples very high values of critical current were achieved, among the best reported to date for commercial 2G HTS wire^7,26,30–34^. In particular, an *I*_*c*_ at 20 K, 20 T in the 220–270 A/4 mm range and an *I*_*c*_ at 4.2 K, 20 T in the 450–570 A/4 mm range were measured. Record *J*_E_ values for commercial wires of over 1000 A/mm^2^ at 20 K, 20 T and over 2000 A/mm^2^ at 4.2 K, 20 T were established for 40 μm substrate with 5 μm per side of stabilising copper. Despite a certain variation in the *I*_*c*_ values in the three samples, there is a low statistical scatter in the ratio of the *I*_*c*_ at 4.2 and 20 K to that at 77 K in self-field (the so-called lift-factor explained in the Methods section), for these samples (Fig. 1a, inset), as well as for the entire production as we show below. This verifies the applicability of the lift-factor approach in the case of this simplified YBCO wire, in contrast to previous reports for other, more complex 2G HTS wire formulations^26,27^.

In Fig. 1b we compare the relative performance of our YBCO and GdBCO^35,36^ wires prepared on the same production line, over a wide range of temperatures and magnetic fields, using a representative *I*_*c*_ at 77 K in self-field of 180 A/4 mm for wires of both types. In the entire range of magnetic fields studied, at temperatures of 65 K and below, YBCO wire outperforms GdBCO wire by a factor of 1.5 to over 2.5. Only at 77 K is GdBCO preferable for application.

**Figure 2:**
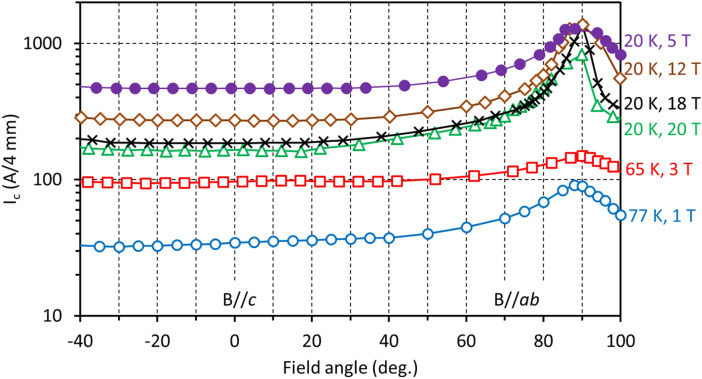
Angular dependences of *I*_*c*_ in magnetic field of YBCO wire at 77 K, 1 T; 65 K, 3 T and 20 K, 5, 12, 18, and 20 T. Measurements were performed at Tohoku University. 0° corresponds to the *B//c* orientation and 90° corresponds to the *B//ab* orientation. Within the accuracy of measurement, the minimum value of *I*_*c*_ for all field orientations is at *B//c*. At 20 K, *I*_*c*_ at *B//ab* stays almost constant with increasing magnetic field. We believe the smaller value of *I*_*c*_ at *B//ab* at 20 T may be an artefact due to the sharp *I*_*c*_ angular peak and the discrete angle step during measurements.

Due to the structural anisotropy of YBCO, *I*_*c*_ depends on the magnetic field direction (Fig. 2). The maximum of *I*_*c*_ occurs with field applied parallel to the wire surface (90°, *B//ab*). Importantly, there is no *I*_*c*_ peak at the 0° (*B//c*) orientation, as is typical for REBCO films with *c*-axis correlated artificial pinning centres^21–25^. In a wide angular region about the *B//c* orientation, the *I*_*c*_ dependence is flat, with the *I*_*c*_ variation below 3%. Therefore, for YBCO wire the minimum *I*_*c*_ for all field orientations, an important parameter for practical use, is at *B//c*.

### Statistical verification of results

**Figure 3:**
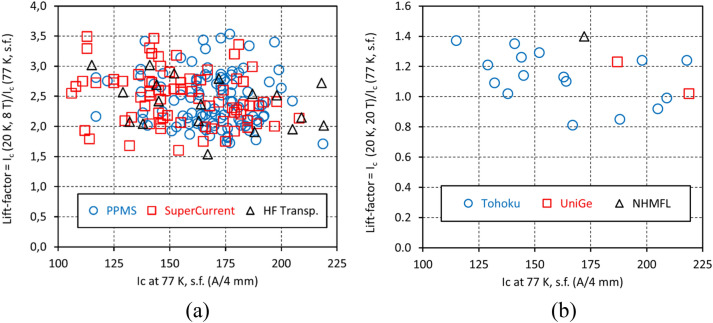
Statistical scatter of lift-factors at (**a**) 20 K, 8 T (*B//c*) and (**b**) 20 K, 20 T (*B//c*) measured by different apparatus. Data points from different measurement apparatus constitute one set. There is no pronounced dependence of the lift-factor value on the *I*_*c*_ at 77 K in self-field. (We provide a table with lift-factor values at 4.2 and 20 K in Supplementary Information).

HTS applications require *reproducible* production at high volume and low cost. Our success is supported by a large and self-consistent data set on samples taken from the front and back ends of the 300–600 m lengths which make up the 300 km of wire underpinning this paper. The samples were evaluated by multiple labs and the finding of identical performance of wire sourced from our two identical production lines in Russia and in Japan. Figure 3 shows the statistical scatter of lift-factors at 20 K determined from measurements with 6 different systems. The data show no pronounced dependence of *I*_*c*_ lift-factor (the ratio of the *I*_*c*_ at 20 K, high field to *I*_*c*_ at 77 K, self-field), even though *I*_*c*_ values ranged quite widely as we developed our ability to make thicker HTS layers^37^.

**Figure 4:**
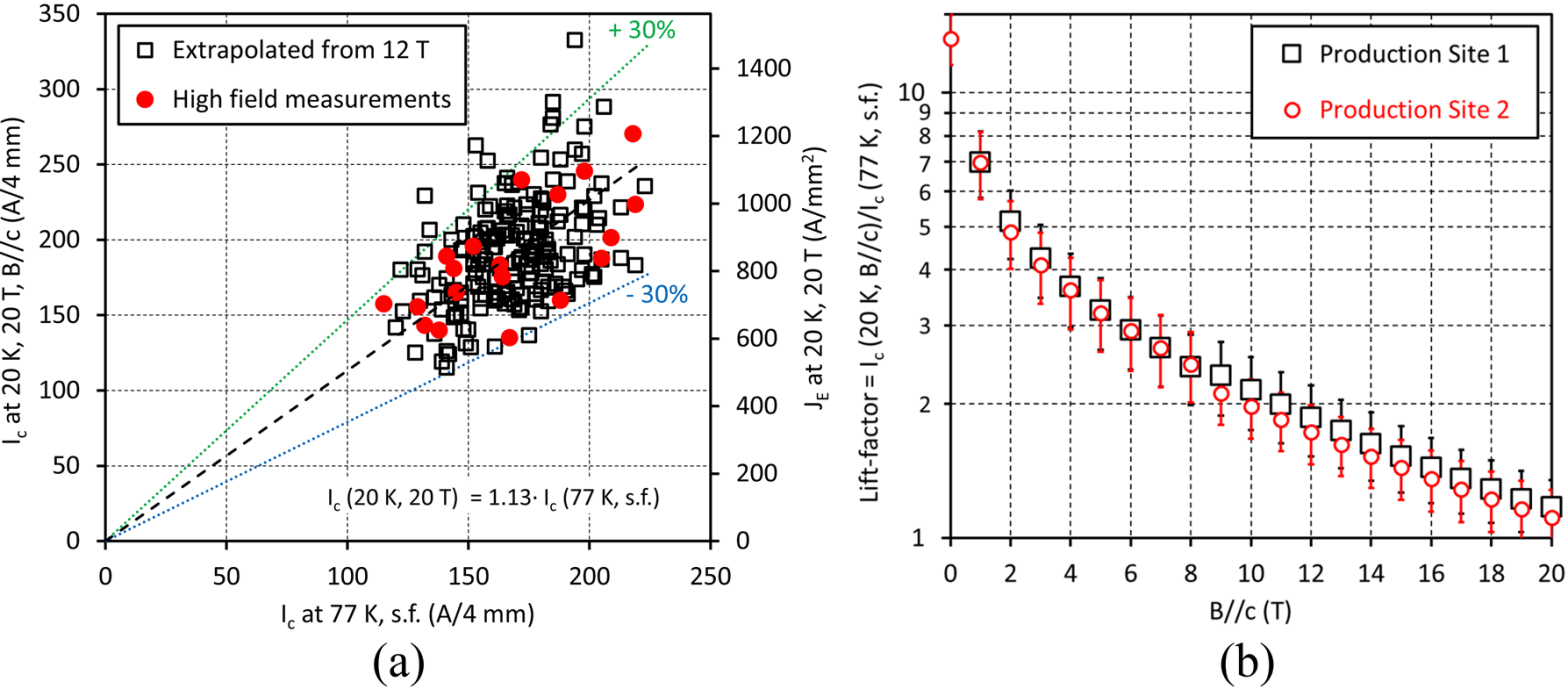
Statistical data for the new YBCO wire at 20 K in magnetic field (*B//c*). (**a**) Correlation between *I*_*c*_ at 77 K in self-field and *I*_*c*_ at 20 K, 20 T. Linear approximation starting at origin gives the slope (lift-factor) of 1.13. Almost all data points lie within the + /− 30% corridor, which is approximately 4 σ. The right vertical axis represents engineering current density for wire on a 40 μm thick substrate with 5 μm per side stabilising copper layer, and a total wire thickness of 56 μm. (**b**) Comparison of lift-factors at 20 K for YBCO wires fabricated at two production sites in Russia and Japan. The lift-factor values almost coincide, well within the statistical spread.

Figure 4a presents the correlation between *I*_*c*_ (77 K, self-field) and *I*_*c*_ (20 K, 20 T). The 200 points cluster tightly along an average “lift-factor” line with an approximately 15% standard deviation. For wires made on a 40 μm thick substrate with 5 μm per side stabilising copper layer, *J*_E_ is in the 500–1400 A/mm^2^ range, 87% of the wires having a *J*_*E*_ above 700 A/mm^2^ and 72% having a *J*_*E*_ of 700–1000 A/mm^2^.

Figure 4b compares lift factors at 20 K for both our Russian and Japanese production lines. The differences are very small and well within the statistical variation. This verification is of high practical importance, though not surprising, because we use identical production equipment at each site. The good reproducibility is due to two main reasons: the simple microstructure of the YBCO layer containing no *c*-axis correlated nano-columns and the reproducible and well-controlled PLD technology used to grow the HTS layer.

## Discussion

We have established a record engineering current density in commercial wire of over 1000 A/mm^2^ at 20 K, 20 T and over 2000 A/mm^2^ at 4.2 K, 20 T. These values surpass the ambitious *J*_*E*_ targets of 700 A/mm^2^ at 20 K, 20 T for fusion magnets^18^ and of 1000 A/mm^2^ at 4.2 K, 20 T for accelerator magnets^12^. This is possible through the combination of a high in-field *I*_*c*_ and a thin substrate. Especially important is that we obtain these extraordinarily high values not in select champion samples but in hundreds of kilometres of routinely manufactured wires. Indeed, this achievement has caused SPARC to raise their specification to 750 A/mm^2^ because of the benefits to the system design.

A common approach to enhance *J*_*c*_ in magnetic field has been to introduce artificial pinning centres (APC) of *c*-axis correlated nano-columns of various perovskites^21–25^, a technique utilised in commercial PLD^28,29,38^ and metalorganic chemical vapour deposition (MOCVD) film growth^6^. Although the highest *J*_*c*_*(B)* values have been obtained in this way^39–41^, the complex HTS film nanostructure results in considerable spread in commercial wire in-field performance^26,27^ and greatly narrows the processing window, requiring slower deposition rates to achieve maximum *J*_*c*_ enhancement^28,29^. For 2G HTS wire with nano-columnar APC, more typical is a faster decay of critical current with increasing magnetic field than for wire without APC^42^. At a temperature of approximately 30 K and below, most pinning occurs on abundant point defects; therefore, APC only indirectly influence the pinning properties by altering the point defect concentration and distribution.

The overall trend to improving HTS wire performance has long been to keep increasing the complexity of the material composition and microstructure by introducing such APC defects, a path which is in direct conflict with nurturing mature, cost-effective mass production technologies. We attribute the great stability of our commercial production to our choice of native Y_2_O_3_ nanoparticles as dominant pinning centres. They do not increase the chemical complexity of YBCO and they impart a simple, uniform nanostructure, amenable to reproducible fabrication.

Indeed, our present *J*_*c*_*(B)* results are superior to many excellent results achieved with nano-columnar APC reported by other groups^7,26,30–34^. We conclude, therefore, that nano-columnar defects are not indispensable for high critical current in magnetic field. Indeed, rare earth oxides form randomly distributed nanoparticles semi-coherent to the REBCO matrix and lead to isotropic enhancement of *J*_*c*_. For instance, Xu et al.^43^ reported a very high pinning force of 1 TN/m^3^ at 4.2 K, 16 T (B//c) in YBCO films with Y_2_O_3_ nanoparticles. It is interesting that the Y_2_O_3_ nanoparticle density of 4000 ± 2000 μm^-2^ in our YBCO films is of the same order of magnitude as the BaZrO_3_ nano-column density in REBCO films in ref.^39^. With the average distance between the Y_2_O_3_ nanoparticles of 16 nm, we calculate matching field of approximately 8 T. The pinning force field dependence (graphs are not presented here) saturates at approximately 15 T at 20 K, but at 4.2 K pinning force keeps increasing beyond 20 T, exceeding 1 TN/m^3^. The values of the α exponent in the power law dependence of *J*_*c*_ ~ *H*^*-α*^ are approximately 0.6 at 20 K and 0.7 at 4.2 K, which is within the typical range for REBCO films and corresponds to the collective pinning large-bundle regime^44^.

Our observations and cited literature indicate that the influence of RE_2_O_3_ nanoparticles on the pinning landscape of REBCO films needs further study, including investigation of the effects of the size, concentration and orientation type of the nanoparticles. We have found two types of axial orientation of Y_2_O_3_ nanoparticles in our YBCO films: (001) and (110), and plan to study their effects on the pinning properties of YBCO and report them in future publications.

**Figure 5:**
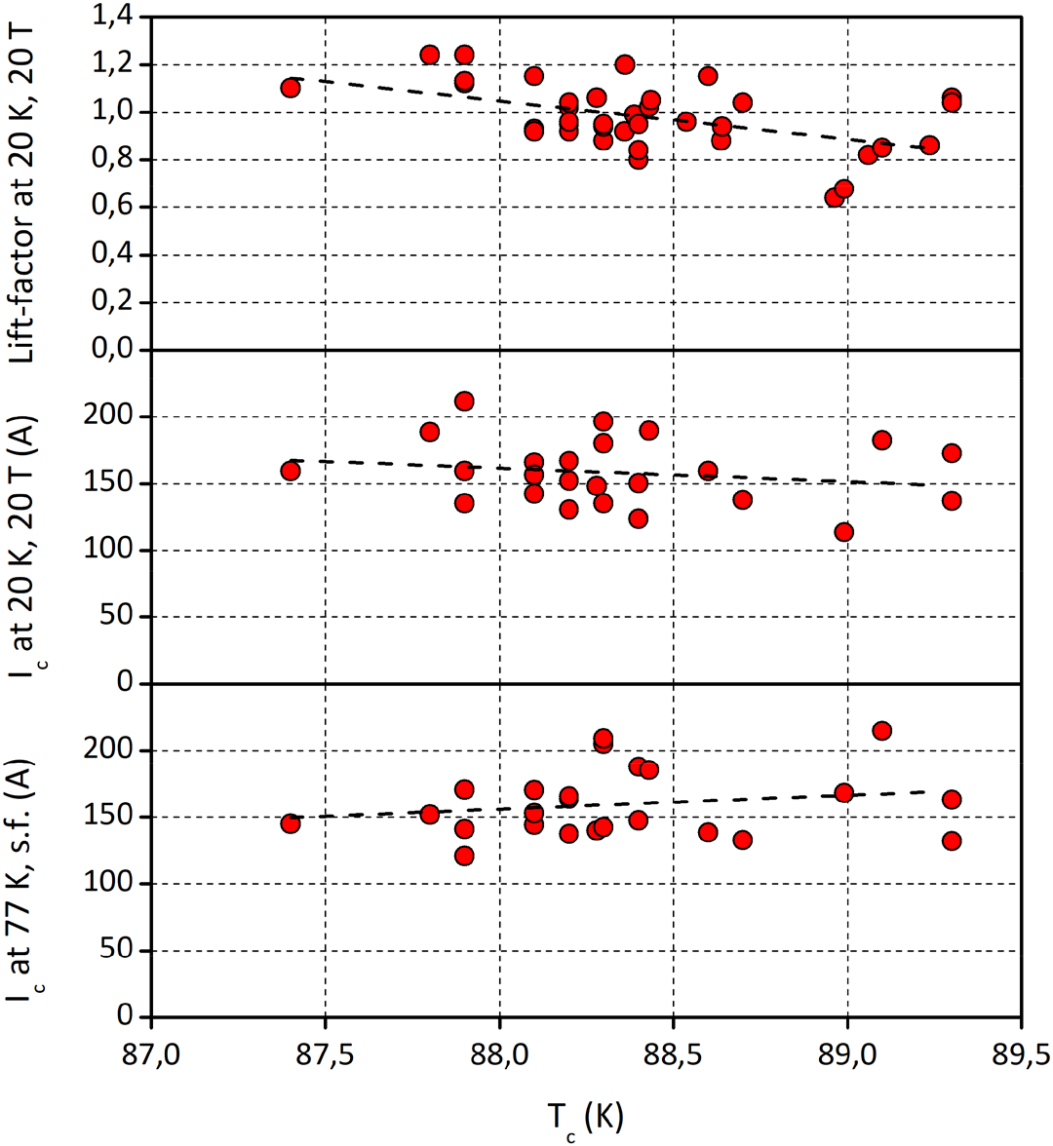
The correlation between the *T*_*c*_ of YBCO films and their superconducting properties at 77 K, self-field and 20 K, 20 T. The higher oxygen content in YBa_2_Cu_3_O_7-δ_ results in overdoping of YBCO with charge carriers and leads to the lower *T*_*c*_ and lower *I*_*c*_ at 77 K in self-field, but to the higher *I*_*c*_ and greater lift-factor values at 20 K, 20 T.

Another route to improving *J*_*c*_*(B)* at low temperature is to enhance the charge carrier (hole) density in the superconductor. High hole doping levels lead to smaller anisotropy and higher irreversibility field^20^. The doping pattern of fully oxygenated REBa_2_Cu_3_O_7-δ_ (δ ≈ 0) has been well studied, and low temperature anneals at high oxygen partial pressure are applied. Recently, Zhang et al.^45^ reported a systematic dependence of REBCO *J*_*c*_*(B)* on the RE^3+^ radius, obtaining higher *J*_*c*_ for the smaller radii. Although the authors did not attribute the effect to the hole doping level, our data comparing YBCO and GdBCO wires (Fig. 1b) made on our production lines support this conclusion, too. The Y^3+^ ionic radius (0.102 nm) is smaller than that of Gd^3+^ (0.105 nm), resulting in CuO_2_ plane hole concentrations of 0.287 and 0.273, respectively^46^. This difference is also manifested in the lower anisotropy of YBCO, with the following values typical for our HTS wires: the YBCO *c*-parameter of 1.170 nm and a superconducting transition temperature, *T*_*c*_, of 88–89 K and the GdBCO *c*-parameter of 1.172 nm and a *T*_*c*_ of 93 K. Thus, we saw a correlation between the *T*_*c*_ of YBCO films and the lift-factor to 20 K, 20 T: the lower the *T*_*c*_–-as manifestation of overdoping with complete oxygenation of YBCO and the oxygen content in YBa_2_Cu_3_O_7-δ_ approaching 7, the higher the lift-factor value (Fig. 5). The same sign of correlation between the *I*_*c*_ at 20 K, 20 T and the *T*_*c*_ suggests that this effect is likely not a normalisation artefact of the lift-factor methodology, which one might suspect due to the reduced *I*_*c*_ at 77 K in self-field in overdoped YBCO films with the lower *T*_*c*_.

It is interesting that GdBCO and YBCO wires in Fig. 1b both contain RE_2_O_3_ nanoparticles^29,36,47^. The density of Gd_2_O_3_ nanoparticles in GdBCO, however, is much lower than that of Y_2_O_3_ nanoparticles in YBCO. We believe this is the manifestation of the differences in YBCO and GdBCO phase diagrams in epitaxial films^48^, and that the resulting difference in microstructure contributes to the difference in in-field properties. It is only at 77 K where the slightly higher *T*_*c*_ of GdBCO offers advantage that it exceeds YBCO wire. At lower temperatures the combination of Y_2_O_3_ nanoparticles and the higher hole doping level of YBCO makes YBCO wires superior.

We have developed a product that satisfies specific performance requirements from the fusion industry, which has created an unprecedented demand on HTS wire. When this demand turns into orders, HTS industry will scale the production driving down the wire cost ultimately to tens of dollars per kiloAmpere-metre, at which level commercial fusion plants become economically feasible^18^, as well as many other commercial HTS applications.

## Conclusion

We have developed 2G HTS wire with a very high performance in magnetic field: engineering current density over 1000 A/mm^2^ at 20 K, 20 T and over 2000 A/mm^2^ at 4.2 K, 20 T. This is a result of our engineering a simple Y_2_O_3_ intrinsic nanoparticle vortex pinning centres in an easy to fabricate and control commercial production environment. The Y_2_O_3_ provides isotropic pinning amplified by point defects arising from the epitaxial strain induced by the presence of the nanoparticles. In addition, the high CuO_2_ plane hole doping level minimises the electronic anisotropy and strengthens the vortex pinning of YBCO. We emphasise that this newly developed YBCO wire is not a research laboratory scale result, but a real commercial product made daily in large quantities and available on the market. The incentive for this innovative industrial development has been market pull from an ambitious project aimed at commercialising fusion power through the use of high field HTS magnets setting unprecedented performance targets for 2G HTS wire.

A simple, robust 2G HTS wire formulation with enhanced performance brings benefits not only to the prospective fusion magnets, but also to all HTS applications. The cost reductions enabled by high-volume, reliable manufacturing of this HTS wire will allow 2G HTS to transition from a “novel” material only used in small quantities by specialised research labs to commercial applications such as energy production, power transmission, grid protection, and MRIs which will be seen by and benefit all of society.

## Methods

### 2G HTS wire fabrication

**Figure 6:**
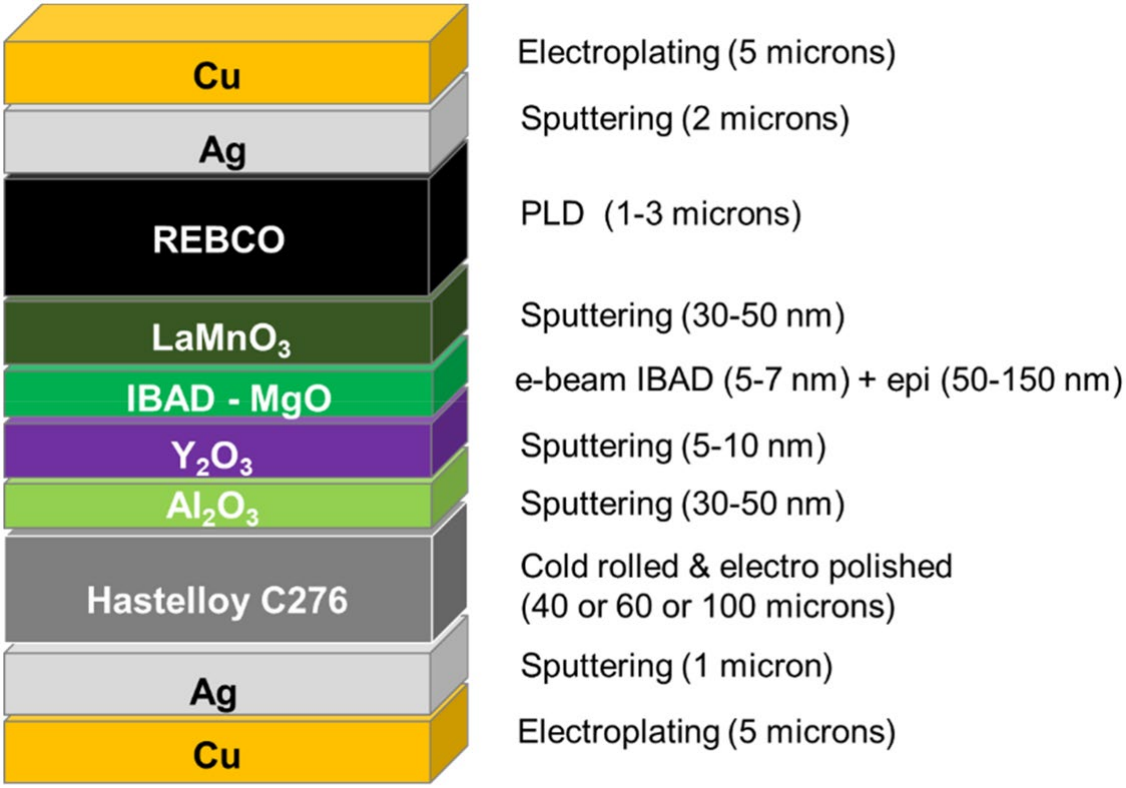
Layer-by-layer structure of 2G HTS wire described in this article.

2G HTS wire was fabricated using production equipment at the SuperOx group of companies: at S-Innovations LLC in Moscow, Russia and at SuperOx Japan LLC in Kanagawa, Japan. The wire architecture is based on cold rolled Hastelloy C276 substrate, biaxially textured MgO buffer layer deposited by ion beam assisted deposition (IBAD), and YBa_2_Cu_3_O_7_ HTS layer deposited by pulsed laser deposition (PLD). The full architecture is given in Fig. 6 and was described in detail elsewhere^35,36,49^.

Wire fabrication starts with a 12 mm wide substrate; if needed, the wire is slit to narrower width after the deposition of the HTS and silver protective layers. Substrate tapes of 100 ± 3 µm, 60 ± 3 µm and 40 ± 3 µm thickness were used in this work.

Substrate electropolishing and buffer layer deposition were performed at S-Innovations, and the HTS layer growth by PLD was performed at S-Innovations and SuperOx Japan, using Coherent LEAP 130C (200 Hz) and LEAP 300C (300 Hz) lasers. Ceramic targets with an excess of yttrium oxide with respect to the stoichiometric YBa_2_Cu_3_O_7_ composition were used, in order to ensure the formation of Y_2_O_3_ phase in the YBCO film matrix.

YBCO film thickness in different wires ranged from 1.5 to 3.5 µm, with an average among all wires of 2.4 µm and a standard deviation of 0.3 µm. Thicker YBCO layers were deposited in order to achieve higher absolute critical current and higher engineering current density. After the YBCO production conditions were established and the superconducting performance was ascertained, the YBCO thickness of about 2.4 µm was chosen as optimal for the combination of high performance and adequate production throughput.

After the YBCO layer deposition, a silver layer was deposited by magnetron sputtering at room temperature, with a thickness of 2 µm on the HTS side and of 1 µm on the substrate side.

Most 12 mm wide wires were mechanically slit to 4 mm width. After slitting, an additional silver layer, at least 1 µm thick, was deposited onto the slit edge, to protect the exposed HTS layer. For electrical stabilisation, surround copper layer, in most cases 5 µm per side, was deposited onto 4 mm wide wires by electroplating.

Production wires were routinely made in 300–600 m lengths, mostly on the 40 μm thick substrate.

### Deposition of thick YBCO films on thin substrate

To further increase *J*_*E*_, we deposited thick YBCO films on a thin (40 μm thick) substrate. Although both these approaches are straightforward and are generally followed for the purpose^6,21,39–41,50,51^, they are nontrivial to implement in economical, high yield production. For thick REBCO films typical is the decay of microstructure and *J*_*c*_ when the film thickness exceeds 1 micron. Certain improvements have been demonstrated by the careful temperature control of the growing film surface. Recently, we have reported high *J*_*c*_ and absolute *I*_*c*_ in more than 3 μm thick GdBCO films by improved temperature profile in the HTS film deposition zone^37^. Here we successfully used the same approach to grow thick YBCO films.

The buffer layer and HTS film quality and superconducting performance were the same on substrates of all thicknesses we used: 100, 60 and 40 microns. However, the production yield was initially lower on the 40 μm thick substrate due to mechanical damage of the tape during winding procedures at all process steps. We resolved this mechanical issue by improving tape tension control in all winding systems. In particular, tension relieve points were added in multipass winding systems.

### Routine characterisation of 2G HTS wire

**Figure 7:**
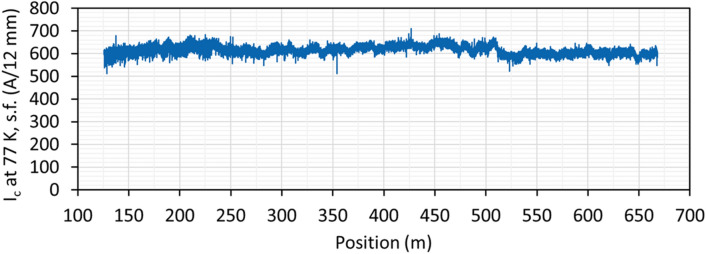
Typical data on critical current at 77 K in self-field along the length of a 542 m long, 12 mm wide YBCO-based 2G HTS wire, serial number 1034 (126–668). Average critical current is 618 ± 18 A.

Positional non-contact measurements of critical current at 77 K in self-field were performed along the entire length of each wire with a TapeStar XL machine, with a longitudinal resolution of 2 mm. As-measured non-contact *I*_*c*_ data for each wire were calibrated by the standard 4-contact transport DC measurements, using a 1 µV/cm criterion for *I*_*c*_. Typical calibrated TapeStar XL data for a production wire are shown in Fig. 7.

The critical current, *I*_*c*_, at 77 K in self-field was 175 A, averaged over the entire wire lot. The best 10% of wires had an *I*_*c*_ of over 200 A. With a typical YBCO layer thickness of 2.35 microns, the average critical current density, *J*_*c*_, was 1.86 MA/cm^2^.

X-ray diffraction analysis was performed with a Rigaku SmartLab diffractometer (CuKα_1_) by measuring θ-2θ-, φ-, and ω-scans. The mean particle size of the Y_2_O_3_ crystallites was calculated using the Scherrer equation.

Surface morphology was analysed with a Carl Zeiss EVO50 scanning electron microscope equipped with an IXRF energy dispersive element analysis system.

YBCO film thickness was determined gravimetrically by weighing three 30 cm long pieces of 12 mm wide wire before and after dissolving the HTS layer in 5% nitric acid^52^.

Delamination energy was routinely tested on samples taken from ends of each 12 mm wide wire in silver finish by the climbing drum method^53^ using a Tinius Olsen material testing machine 5 ST series and a custom climbing drum rig. Average delamination energy is 7.4 ± 2.5 J/m^2^. Delamination takes place at the buffer layer/HTS layer interface and/or within the buffer layer stack. The reported values of delamination energy and its rather wide variation, as well as failure interfaces are typical for 2G HTS wire industry^53^. We plan to report the delamination measurements and results in detail in a separate publication.

### Transmission electron microscopy

Transmission electron microscopy (TEM) images were taken in an Osiris TEM/STEM (Thermo Fisher Scientific) equipped with a high angle annular dark field (HAADF) electron detector (Fischione) and Bruker energy-dispersive X-ray microanalysis (ERA) system (Bruker) at an accelerating voltage of 200 kV. Image processing was performed using Digital Micrograph (Gatan) and TIA (ThermoFisher Scientific) software. Samples for TEM were prepared using the focused ion beam (FIB) technique in a Versa (ThermoFisher Scientific) dual beam microscope.

### Measurement of critical current in magnetic field

A number of independent measurement techniques and apparatus were used for the measurement of critical current in magnetic field.

A PPMS-9 system at P.N. Lebedev Physics Institute was used for the measurement of magnetisation hysteresis loops in the 4.2–77 K temperature range and 0–8 T (*B*//*c*) magnetic field range. Sample size was 3 × 3 mm. Lift-factors were calculated as ratios of magnetisation at corresponding temperature and magnetic field to that at 77 K, 0 T.

A SuperCurrent system^54^ at Robinson Research Institute was used for transport current measurements of *I*_*c*_ in the 82.5–20 K temperature range and 0–8 T field range (*B//c* and angular dependences) on full 4 mm width samples.

A SuperCurrent system at Commonwealth Fusion Systems was used for transport current measurements of *I*_*c*_ in the 77–20 K temperature range and 0–12 T field range (*B//c* and angular dependences) on full 4 mm width samples. The dependence of critical current on magnetic field at 20 K was extrapolated to 20 T using the fit equation published in^55^.

The *I*_*c*_*(B, T, Ɵ)* data collection in High Field Laboratory for Superconducting Materials at Tohoku University was carried out using 30 µm and 40 µm bridges of 1 mm length fabricated by picosecond laser micromachining from the 4 mm tapes with the top Ag layer. The measurements were performed at 77, 65, 40, 20 and 4.2 K using 20 T-CSM^56^ and 25 T-CSM^57^ cryogen-free superconducting magnets. The angular dependence *I*_*c*_*(Ɵ)* data were collected in the range from − 45° to 120°.

High-field measurements at NHMFL were performed on full 4 mm width samples in two magnets. For in-field experiments up to 15 T, we used the Oxford Instruments 15 T/17 T magnet system with a 52 mm cold bore. Samples were immersed in liquid helium during experiments at 4.2 K. Samples were in helium gas during experiments at 20 K^26^ In experiments up to 31.2 T we used the NHMFL resistive magnet system (cell 7) with a 50 mm bore magnet; 38 mm in Janis cryostat. More experimental detail can be found in^38^.

The experimental setup at the University of Geneva allows measuring *I*_*c*_ up to 2 kA at 4.2 K in liquid He and up to 1 kA in He gas flow by standard four-probe measurement. A 19 T (at 4.2 K) / 21 T (at 2.2 K) superconducting solenoid magnet from Bruker BioSpin completes the system. A temperature precision down to ± 0.01 K is achieved in He gas flow up to 50 K using an active temperature stabilisation system which compensates the heating during current runs with PID controlled heaters^58^.

### Lift-factor methodology

We used the so-called “lift-factor” methodology in our result analysis. This methodology is accepted among 2G HTS wire manufacturers^27,28,36^; however, it has certain applicability constraints and thus is often criticised^26,27^. Lift-factor is a simple empirical *I*_*c*_ scaling parameter: it is defined as the ratio of a sample’s *I*_*c*_ at a specific temperature and magnetic field to the *I*_*c*_ of the same sample at 77 K in self-field. If lift-factors reproduce reasonably well among many different samples of the same type (same HTS layer chemical composition, growth conditions, etc.), a database of previously measured lift-factors (see Table 1 in Supplementary Information and ref.^59^) becomes a useful predictive tool for the estimation of the wire performance in specific operation conditions: one just needs to measure the wire’s critical current at 77 K in self-field and multiply it by the corresponding lift-factor.

## Supplementary Information

Supplementary Information
