# Supplementary material for: Development and large volume production of extremely high current density YBa2Cu3O7 superconducting wires for fusion
Source: Sci Rep. 2021 Jan 22;11:2084. doi: 10.1038/s41598-021-81559-z (PMC7822827; doi:10.1038/s41598-021-81559-z)
Supplement: Supplementary file 1 — Supplementary Information [file 41598_2021_81559_MOESM1_ESM.docx]

**Development and large volume production of extremely high current density YBa_2_Cu_3_O_7_ superconducting wires for fusion**

A. Molodyk* ^1, 2^, S. Samoilenkov^1, 2^, A. Markelov^1^, P. Degtyarenko^2, 3^, S. Lee^4^, V. Petrykin^4^, M. Gaifullin^4^, A. Mankevich^1^, A. Vavilov^1, 2, 4^, B. Sorbom^5^, J. Cheng^5^, S. Garberg^5^, L. Kesler^5^, Z. Hartwig^6^, S. Gavrilkin^7^, A. Tsvetkov^7^, T. Okada^8^, S. Awaji^8^, D. Abraimov^9^, A. Francis^9^, G. Bradford^9^, D. Larbalestier^9^, C. Senatore^10^, M. Bonura^10^, A. E. Pantoja^11^, S. C. Wimbush^11^, N. M. Strickland^11^, A. Vasiliev^12, 13, 14^

1 S-Innovations, Moscow, Russia

2 SuperOx, Moscow, Russia

3 Joint institute for High Temperature, Russian Academy of Sciences, Moscow, Russia

4 SuperOx Japan, Kanagawa, Japan

5 Commonwealth Fusion Systems, Cambridge MA, USA

6 Massachusetts Institute of Technology, Cambridge MA, USA

7 P.N. Lebedev Physics Institute, Russian Academy of Sciences, Moscow, Russia

8 Institute for Materials Research, Tohoku University, Sendai, Japan

9 National High Magnetic Field Laboratory, Florida State University, Tallahassee FL, USA

10 University of Geneva, Geneva, Switzerland

11 Robinson Research Institute, Victoria University of Wellington, New Zealand

12 National Research Centre “Kurchatov Institute”, Moscow, Russia

13 Shubnikov Institute of Crystallography, Russian Academy of Sciences, Moscow, Russia

14 Moscow Institute of Physics and Technology, Dolgoprudny, Russia

* corresponding author: Alexander Molodyk, e-mail: a.molodyk@superox.ru

**Supplementary Information**

*Microstructure analysis*

An image of the surface morphology of a typical YBCO film is shown in Supplementary Figure 1 (a). We can observe a dense YBCO film with inclusions of (100)- and (010)-oriented elongated YBCO grains and few precipitates of secondary phases.

XRD analysis confirms a sharp biaxial texture of the YBCO film with an average in-plane orientation FHWM(103) of 2-2.5^o^, out of plane orientation FWHM(005) of 1-1.5^o^ and the YBCO *c* parameter of 1.170 nm. The epitaxial relations in the heterostructure are as follows: [100](001)YBa_2_Cu_3_O_7_// [100](001)LaMnO_3_//[100](001)MgO. This means that the YBCO crystallites are aligned with their (001) planes parallel to the wire surface and either (100) or (010) planes parallel to the wire edge. The films contain a small fraction of (103)-, (013)-, (100)- and (010)-oriented YBCO grains, which are mostly located at or near the film surface, according to electron microscopy observations.

YBCO films contain yttrium oxide particles in the (100)- and (110)-orientation (Supplementary Figure 1 (b)). The mean size of the Y_2_O_3_ particles with the (100)-orientation in the direction normal to the wire surface is 20 ± 2 nm, as calculated from the XRD peak broadening. Based on the XRD data, we could not obtain reliable values for the mean size of the (110)-oriented Y_2_O_3_ particles because of the very low intensity of the (440) Y_2_O_3_ XRD peak. The Y_2_O_3_ lattice parameter in the (100)-oriented inclusions is 1.055 nm, which is 0.6% lower than the bulk value of 1.061 nm. This is the manifestation of the compressive stress applied by the YBCO matrix on the Y_2_O_3_ nanoparticles [60].


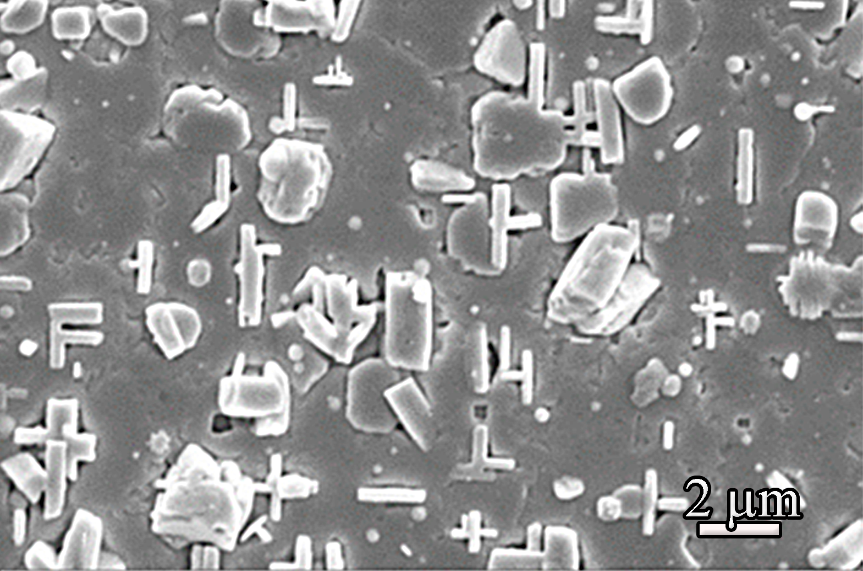
 (a)


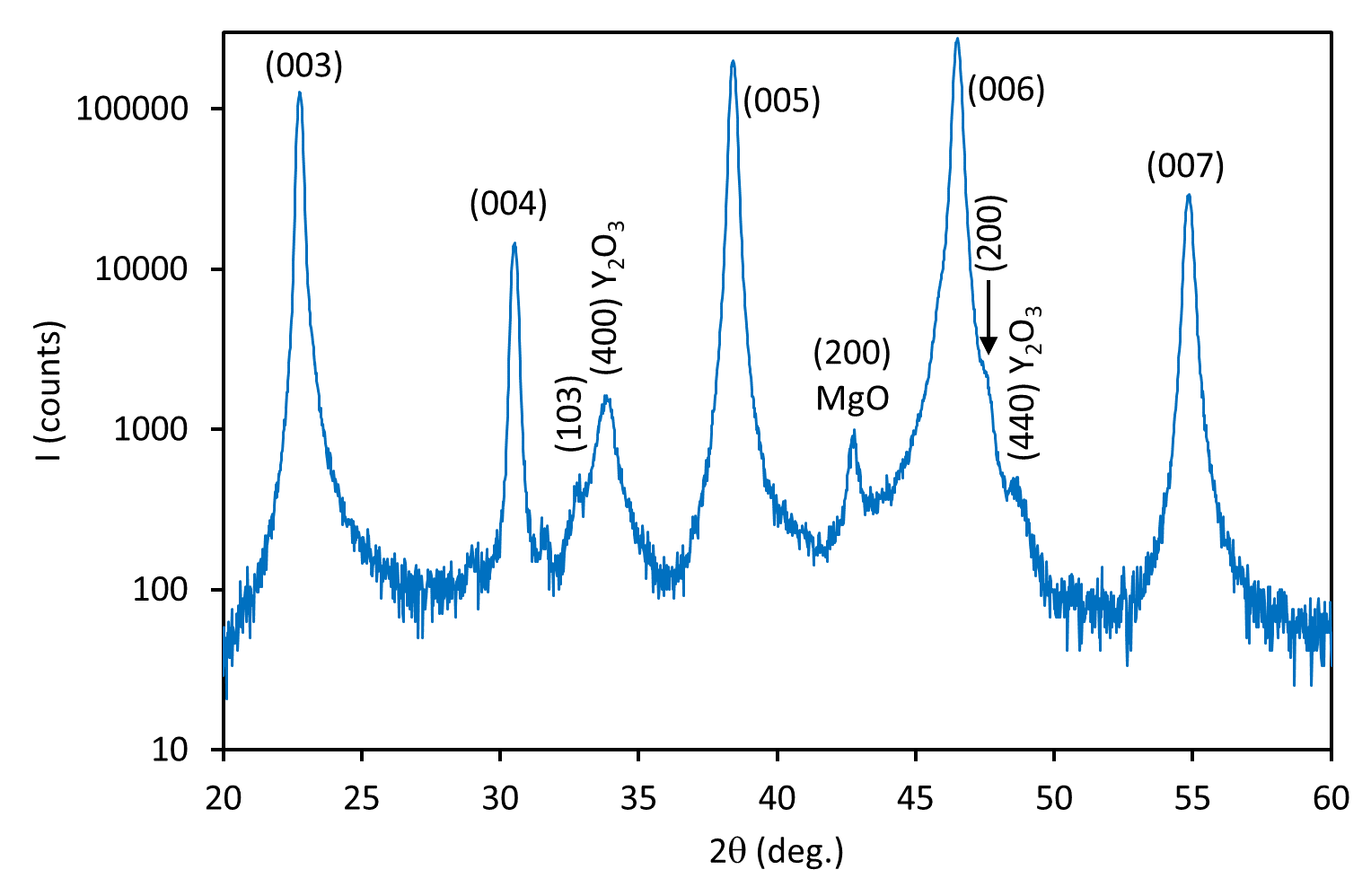
 (b)

Supplementary Figure 1. (a) SEM image of a YBCO film surface. The elongated rectangular particles are the (100)- and (010)-oriented YBCO grains; the particles with the smaller aspect ratio are secondary phase inclusions. (b) θ-2θ X-ray diffraction scan of a YBCO wire showing the (001)-preferred out-of-plane YBCO texture and low intensity peaks of misoriented YBCO grains and Y_2_O_3_ nanoparticles.

Transmission Electron Microscopy (TEM) reveals dense YBCO films with an average thickness of about 2400 nm. Cross-sectional high-resolution TEM imaging (Supplementary Figure 2) shows a high concentration of semi-coherent Y_2_O_3_ nanoparticles embedded into the YBCO film matrix; the following two orientations were established by the fast Fourier transform analysis: [100](001)Y_2_O_3_//[110](001)YBCO and [001](110)Y_2_O_3_//[010](001)YBCO; this agrees with our XRD results. The (110)-oriented yttria particles have a very small size in the 5-20 nm range and are of a relatively isotropic shape (Supplementary Figure 2 (b)). The (100) particles are platelet-shaped, with 20-100 nm lateral size and 5-30 nm vertical size. In high magnification images, the particles appear randomly distributed, while at a lower magnification some of them seem to be arranged in arrays slightly tilted with respect to the (001)YBCO plane. Average nanoparticle density through the film thickness is 4000 ± 2000 μm^-2^.

The observed microstructural features are typical for REBCO films with nano-inclusions of rare earth oxides [27, 43, 48, 60, 61].

| 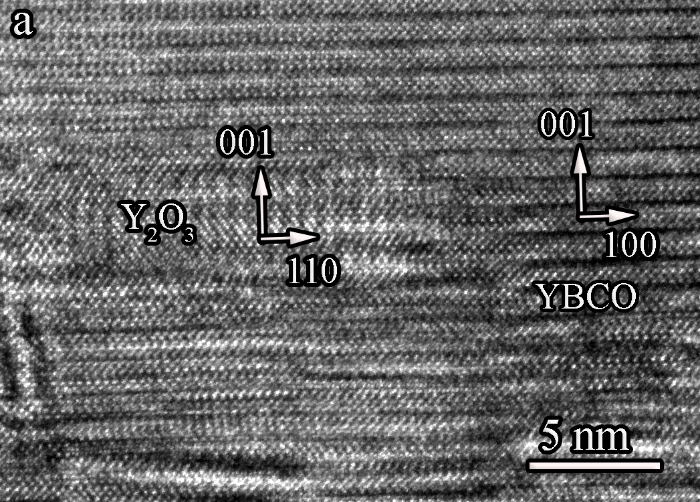 | 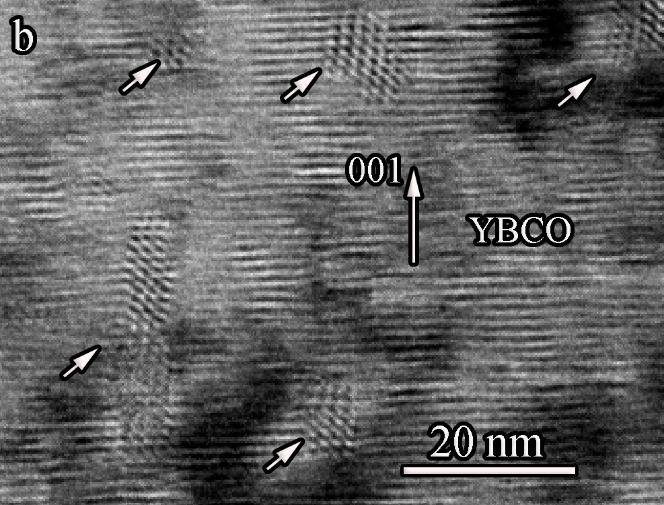 |
| --- | --- |

Supplementary Figure 2. Bright field TEM images showing semi-coherent Y_2_O_3_ nanoparticles in the YBCO matrix. (a) Platelet-shaped (100)-oriented Y_2_O_3_ nanoparticle. (b) (110)-oriented Y_2_O_3_ nanoparticles (arrowed). Particles of this orientation type are always observed as moiré features, which suggests their relatively low aspect ratio with a very small size in the 5-20 nm range.

Supplementary Table 1. Average lift-factors at 4.2 and 20 K for the newly developed YBCO wire.

| *B//c* (T) | Average lift-factor at 4.2 K ± StD | Average lift-factor at 20 K ± StD |
| --- | --- | --- |
| 0 | 17.50 ± 2.13 | 13.23 ± 1.72 |
| 1 | 11.69 ± 1.63 | 6.96 ± 1.19 |
| 2 | 8.79 ± 1.24 | 4.93 ± 0.86 |
| 3 | 7.32 ± 1.04 | 4.14 ± 0.76 |
| 4 | 6.52 ± 0.91 | 3.61 ± 0.66 |
| 5 | 5.76 ± 0.80 | 3.21 ± 0.59 |
| 6 | 5.27 ± 0.73 | 2.92 ± 0.54 |
| 7 | 4.93 ± 0.67 | 2.67 ± 0.49 |
| 8 | 4.60 ± 0.60 | 2.45 ± 0.44 |
| 9 | 4.32 ± 0.56 | 2.21 ± 0.38 |
| 10 | 4.07 ± 0.51 | 2.05 ± 0.35 |
| 11 | 3.85 ± 0.48 | 1.92 ± 0.32 |
| 12 | 3.64 ± 0.45 | 1.79 ± 0.30 |
| 13 | 3.46 ± 0.43 | 1.68 ± 0.27 |
| 14 | 3.29 ± 0.39 | 1.57 ± 0.26 |
| 15 | 3.13 ± 0.36 | 1.48 ± 0.24 |
| 16 | 3.00 ± 0.34 | 1.40 ± 0.22 |
| 17 | 2.86 ± 0.31 | 1.32 ± 0.21 |
| 18 | 2.75 ± 0.31 | 1.25 ± 0.20 |
| 19 | 2.64 ± 0.28 | 1.19 ± 0.16 |
| 20 | 2.55 ± 0.27 | 1.13 ± 0.17 |

**References**

60. S V Samoilenkov, O V Boytsova, V A Amelichev, A R Kaul “Anisotropic strain of BaZrO_3_, BaCeO_3_ and Y_2_O_3_ nanoinclusions in a YBa_2_Cu_3_O_7−x_ epitaxial film matrix and its relation to the oxygen content of the superconductor” 2011 Supercond. Sci. Technol. 24 055003

61. K. Verbist, A. L. Vasiliev, and G. Van Tendeloo, Y_2_O_3_ inclusions in YBa_2_Cu_3_O_7-δ_ thin films, Appl. Phys. Lett. 66, 1424 (1995)
